# Large language models as assistance for glaucoma surgical cases: a ChatGPT vs. Google Gemini comparison

**DOI:** 10.1007/s00417-024-06470-5

**Published:** 2024-04-04

**Authors:** Matteo Mario Carlà, Gloria Gambini, Antonio Baldascino, Francesco Boselli, Federico Giannuzzi, Fabio Margollicci, Stanislao Rizzo

**Affiliations:** 1 Ophthalmology Department, Fondazione Policlinico Universitario A. Gemelli, IRCCS, 00168 Rome, Italy; 2 Ophthalmology Department, Catholic University “Sacro Cuore,”, Largo A. Gemelli, 8, Rome, Italy

**Keywords:** Large language models (LLM), ChatGPT, Google Gemini, Google Bard, Glaucoma, Artificial intelligence (AI), Glaucoma surgery

## Abstract

**Purpose:**

The aim of this study was to define the capability of ChatGPT-4 and Google Gemini in analyzing detailed glaucoma case descriptions and suggesting an accurate surgical plan.

**Methods:**

Retrospective analysis of 60 medical records of surgical glaucoma was divided into “ordinary” (*n* = 40) and “challenging” (*n* = 20) scenarios. Case descriptions were entered into ChatGPT and Bard’s interfaces with the question “What kind of surgery would you perform?” and repeated three times to analyze the answers’ consistency. After collecting the answers, we assessed the level of agreement with the unified opinion of three glaucoma surgeons. Moreover, we graded the quality of the responses with scores from 1 (poor quality) to 5 (excellent quality), according to the Global Quality Score (GQS) and compared the results.

**Results:**

ChatGPT surgical choice was consistent with those of glaucoma specialists in 35/60 cases (58%), compared to 19/60 (32%) of Gemini (*p* = 0.0001). Gemini was not able to complete the task in 16 cases (27%). Trabeculectomy was the most frequent choice for both chatbots (53% and 50% for ChatGPT and Gemini, respectively). In “challenging” cases, ChatGPT agreed with specialists in 9/20 choices (45%), outperforming Google Gemini performances (4/20, 20%). Overall, GQS scores were 3.5 ± 1.2 and 2.1 ± 1.5 for ChatGPT and Gemini (*p* = 0.002). This difference was even more marked if focusing only on “challenging” cases (1.5 ± 1.4 vs. 3.0 ± 1.5, *p* = 0.001).

**Conclusion:**

ChatGPT-4 showed a good analysis performance for glaucoma surgical cases, either ordinary or challenging. On the other side, Google Gemini showed strong limitations in this setting, presenting high rates of unprecise or missed answers.

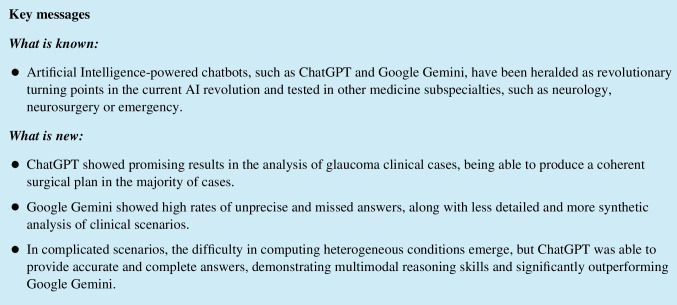



## Introduction

Artificial intelligence (AI) natural language processing has witnessed a significant transformation with the advent of advanced large language models (LLMs) [1]. Two of the most important LLM models are Chat Generative Pretrained Transformer (ChatGPT, created by OpenAI, San Francisco, CA, USA) and Google Gemini (formerly Google Bard, Google, Mountain View, CA). Recently, the role of these models in medical settings has been explored [2].

In the ophthalmological panorama, ChatGPT has shown good results in comprehending clinical information and generating appropriate replies [3, 4]. ChatGPT models are trained on a textual database and are able to produce responses that are both coherent and contextually appropriate, based on the abstract relationship between words (tokens) within the neural network [1]. The capabilities of this LLM in answering to multiple-choice questions from the US Medical Licensing Examination (USMLE) were studied, and it was shown that ChatGPT not only answered properly to more than half of the questions, but also supplied good supporting justifications for the chosen options [5]. Additional features and restrictions of ChatGPT in ophthalmology have been discussed in literature [6].

On March 21, 2023, Google debuted its AI chatbot, Google Bard, an AI chatbot, which uses machine learning and natural language processing to simulate human-like dialogue. Bard can be accessible on several digital platforms, providing accurate answers and assistance in fields such as public health and disaster relief [7]. In the recent months, some research compared the performance of Google Bard to those of ChatGPT in other medicine subspecialties, such as neurology, neurosurgery, or emergency [8–10]. Recently, on February 8, 2024, Google presented the update to a new AI-based platform, called Gemini, embracing optimized features and enhancing multimodal analysis [11].

In a glaucoma setting, several research showed the efficacy of AI models [12–14]. Globally, glaucoma is a prevalent cause of permanent blindness, with a unique modifiable risk factor: intraocular pressure (IOP) [15–17]. Current treatment options include pharmacologic topical therapies reducing aqueous humor production or increasing its outflow, lasers targeting the trabecular meshwork and ciliary body, and surgical treatment [18]. The latter includes a variety of techniques, starting from the gold standard trabeculectomy to the newly developed microinvasive glaucoma surgeries (MIGS) and, in more challenging cases, glaucoma draining devices (GDDs). A tailored approach to surgery, including the search for risk factors for surgical failure, is now required to ensure a long-term IOP control. In order to use the optimum approach, certain risk variables, including age, race, conjunctival status, and past medical or surgical procedures, must be addressed specifically for each patient [19].

The aim of this study was to define the capability of two different LLMs (ChatGPT 3.5 and Google Gemini) in analyzing detailed case descriptions in a glaucoma setting and suggesting the best possible surgical choice. In order to evaluate the accuracy of the two chatbots, the answers were compared to glaucoma specialists’ responses and the level of agreement between them was assessed.

## Materials and methods

### Case collection

We conducted a retrospective review of the files of glaucomatous patients who underwent any type of surgical or para-surgical glaucoma treatment in Policlinico Universitario Agostino Gemelli between May 2019 and October 2023. This study adhered to the tenets of the Declaration of Helsinki, and the Institutional review board (IRB) approval was obtained from the Policlinico Universitario Agostino Gemelli Institutional Ethics Committee.

We selected a total of 66 medical records from a pool of over 250. Patient demographics and medical or ocular anamnesis, actual ocular diseases and topical medication, referred symptoms, and examination results were all described in detail in each instance. Finally, this information was summarized in a complete case description and submitted at the same time to the ChatGPT and Google Gemini interfaces.

Primary open-angle glaucoma, normal-tension glaucoma, primary angle-closure glaucoma, pseudoexfoliation glaucoma, pigment dispersion glaucoma, glaucomatocyclitic crisis glaucoma, aphakic glaucoma, neovascular glaucoma, and uveitic glaucoma were among the phenotypes represented.

Successively, we separated the mentioned instances into two categories one by one:

1. “Ordinary” scenarios (*n* = 42): patients who had no previous eye surgery other than cataract surgery and no severe concurrent illnesses or ocular disorders. Only topical medication and/or prior laser treatments were used to control IOP.
2. “Challenging” scenarios (*n* = 24): individuals with a considerably more complicated medical history, including past ocular operations such as unsuccessful glaucoma procedures, vitreoretinal surgeries, anterior segment surgeries, or other ocular diseases influencing the eye’s homeostasis. Furthermore, instances with concurrent systemic illnesses that may have influenced surgical decisions were included in this sample.

### ChatGPT

Built on the “gpt-3.5-turbo” model from the GPT-3.5 series, ChatGPT (OpenAI, https://chat.openai.com/) is an optimized LLM. Up to September 2021, billions of text data taken from online articles were used to train Generative Pretrained Transformer 3 [1]. The model is trained to minimize the difference between the predicted and actual words in the training data set. After the model is trained, fresh text may be produced by giving it an instruction and letting it guess what word will come next. The process is then repeated until the model generates a whole sentence or paragraph, using the predicted word as the background for the next prediction [20]. In comparison to its predecessor, the most recent version, GPT-4, was recognized for having more accuracy and efficiency. The main breakthroughs were increased knowledge base, better language proficiency, and better contextual comprehension [21].

### Google Gemini AI

Google Bard AI (Google, https://bard.google.com/chat/) is built on the Pathways Language Model 2 (PaLM 2), a massive language model designed to be very effective at understanding facts, logical thinking, and arithmetic [22]. It is able to respond to a broad range of user queries and prompts by simulating human-like interactions, providing also thorough and educational answers to user inputs after being trained on a large corpus of text data [22]. Due to its integration with Google’s vast search network, Bard AI has access to up-to-date online data [23].

Gemini 1.0 is a new AI model built in three various sizes (Ultra, Pro, and Nano); a result of extensive collaborative efforts of the Google Team, was announced by the Google Team on December 6, 2023, marking a major platform upgrade. With plans to soon include multimodal reasoning skills, Gemini Pro (Google, https://gemini.google.com/app/) is the first model that is now available and can respond to text-based cues [24].

Although Gemini and ChatGPT are comparable in several ways, Gemini has a remarkable aptitude for comprehending and responding to inquiries that need for exact details, while, on the other hand, ChatGPT may effectively produce a wide range of imaginative and varied texts, including articles, scripts, code, and more, all of which may or may not be entirely correct [22].

### Chatbot-aided surgical choice

**Fig. 1:**
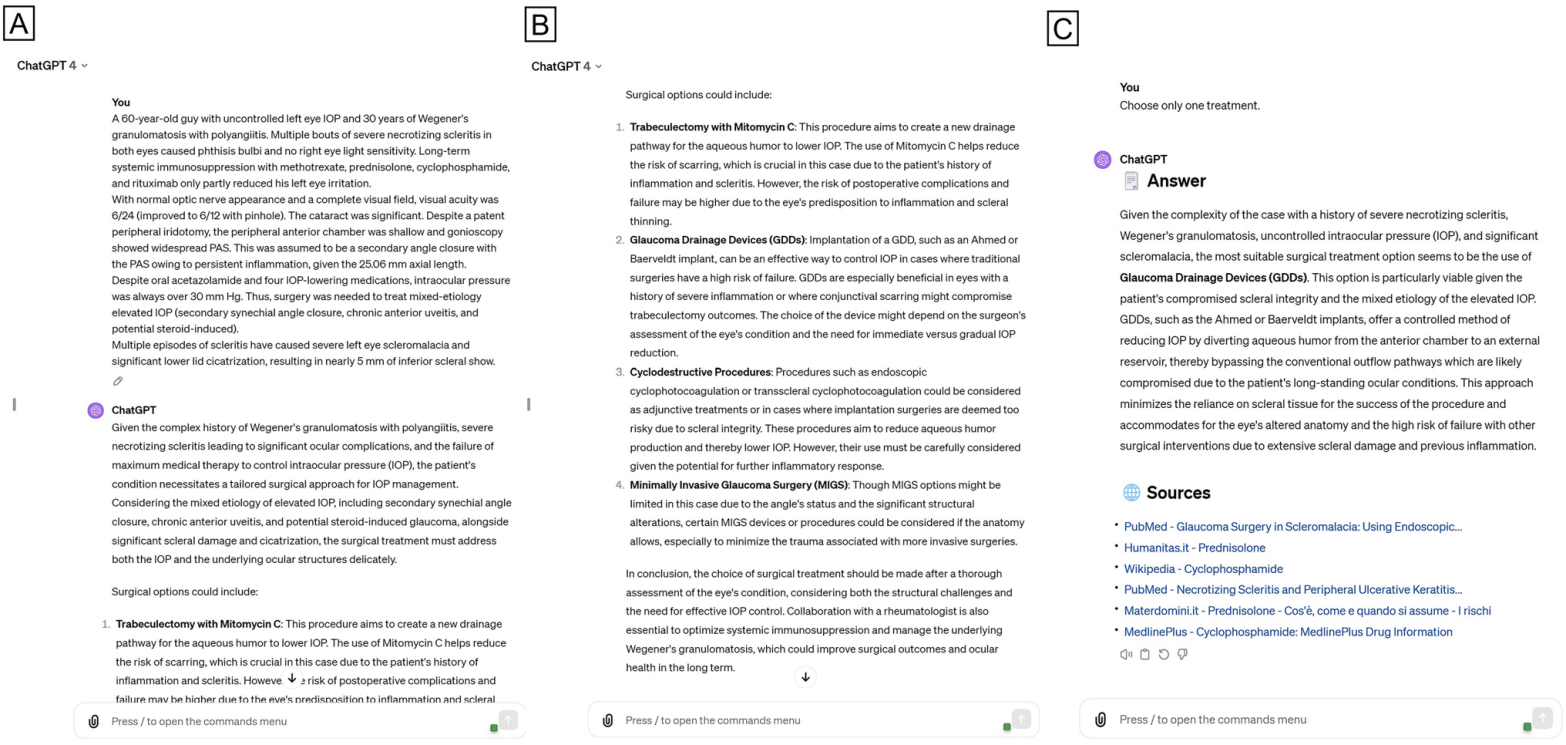
Screenshot of ChatGPT-4 responses in a “challenging” case. **A**, **B** Case description and ChatGPT answer analyzing the scenario and proposing several surgical treatments; **C** coherent answer when asked to choose only one among proposed treatments

**Fig. 2:**
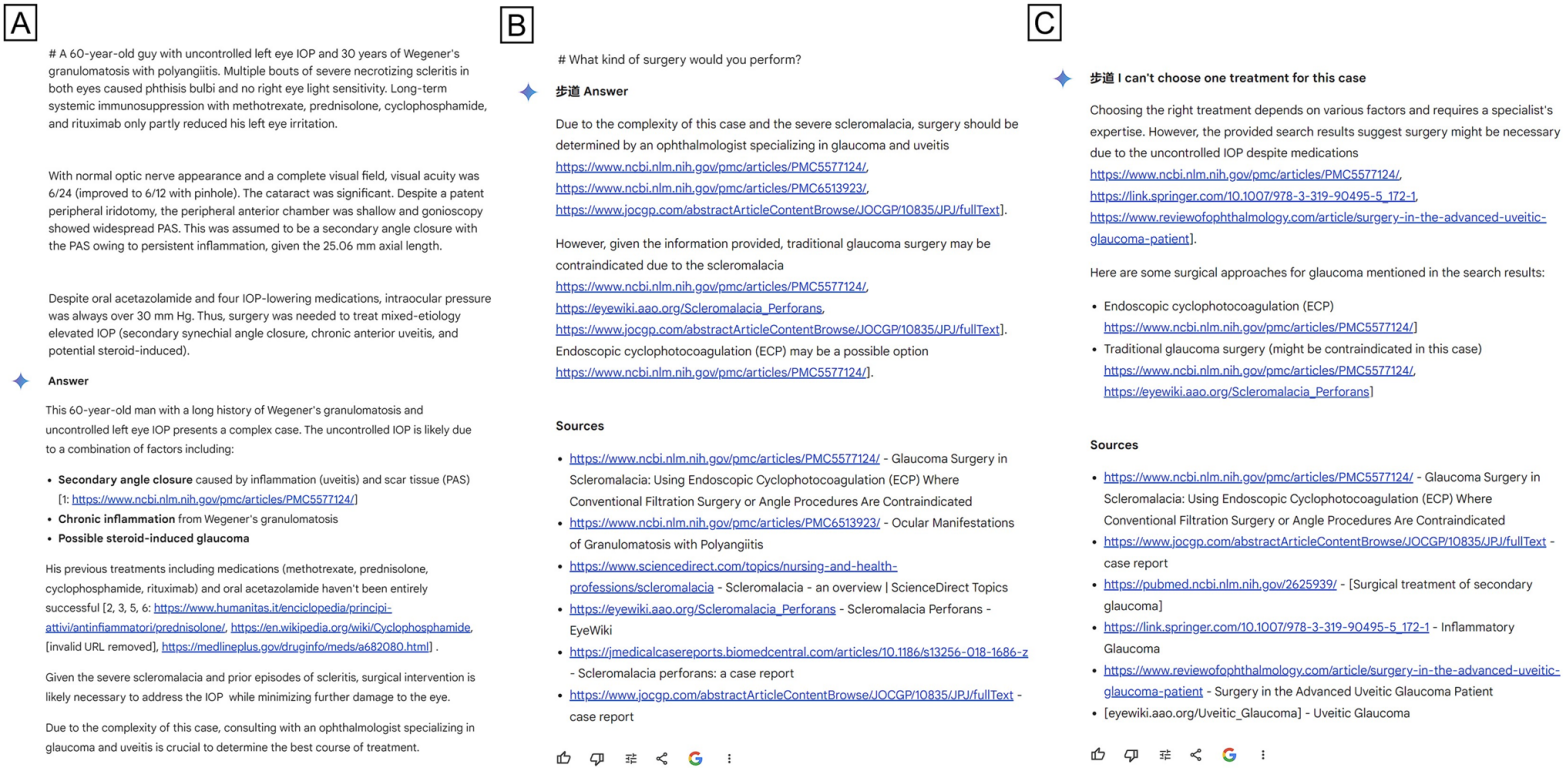
Screenshot of Google Gemini responses in the same “challenging” case of Fig. 1. **A** Case description and Gemini analysis of the case; **B** when asked for surgical advice, Google Gemini provided more synthetic answers rich of web sources; **C** when asked to choose only one treatment, Gemini frequently answered “I can’t choose one treatment for this case.” However, it was able to present a list of surgical options, even though none of them was analyzed in details

We entered every case description into either ChatGPT (version 3.5) or Google Gemini interface. Both platforms were accessed on March 8 and 9, 2024. Firstly, we left the LLM analyze the entire case and highlight key findings. Successively, we investigated about the model’s ability to provide coherent surgical choices, writing the question “What kind of surgery would you perform?” (Figs. 1 and 2). In some instances, when the answers were overly generalist, we asked the model for further explanations regarding the treatment of choice, based on the specific conditions of the clinical case, asking the chatbots to choose only one treatment. We logged all of the comments based on our initial inquiry about provisional and differential surgical choices so that the two chatbots might learn from the past. Finally, in order to assess chatbots’ answers consistency, all questions were repeated three times, and possible changes in surgical suggestions were collected.

In addition, three senior glaucoma specialists (G.G., A.B., and S.R.) were asked to analyze the same 66 cases and to define a shared surgical choice. Cohen coefficient analysis was used to ensure that the concordance was at least 0.90. If all three operators agreed, the ideal surgical treatment for that specific case was identified. Otherwise, the experts discussed the case, and if a univocal choice could not be reached, the case was excluded from the study. Since it was impossible to obtain a comprehensive scenario outline, six patients with incomplete medical histories or anamnesis, missing preoperative data or surgical records on the computer system, or receiving surgery straight from the emergency department were excluded from the study. In fact, the lack of complete pre-operative data raised doubts between the specialists and determined inconsistent results derived from chatbots’ answers.

A correct agreement between chatbots and specialists was considered real only if the chosen treatment of both were the same. If the chatbot was not able to pick one treatment among the presented list, but the answer was indeed present in the list, we defined it as “partial agreement.” In the remaining cases, the answer was considered incorrect. At the end of the dialogue with ChatGPT and Gemini, the 7 ophthalmologists included in this research graded the quality of the answers provided by the two chatbots from 1 (poor quality) to 5 (excellent quality), according to the Global Quality Score (GQS) [25].

As the main outcome of this research, we wanted to define the frequency of accurate surgical choice made by ChatGPT and Google Gemini, compared to those of glaucoma specialists facing the same clinical cases. As secondary outcome, we compared the GQS of the two chatbots.

### Global Quality Score

The GQS, introduced by Bernard et al., is a subjective rate of the overall quality of each web site [25]. It consists in a 5-point Likert scale, taking into account the flow and ease of use of each web site [25]. The complete scale is visible in Table 1.

**Table 1:** Description of the Global Quality Score 5-point scale for web site evaluation

Global score	Global score description
1	Poor quality, poor flow of the site, most information missing, not at all useful for patients
2	Generally poor quality and poor flow, some information listed but many important topics missing, of very limited use to patients
3	Moderate quality, suboptimal flow, some important information is adequately discussed but others poorly discussed, somewhat useful for patients
4	Good quality and generally good flow, most of the relevant information is listed, but some topics not covered, useful for patients
5	Excellent quality and excellent flow, very useful for patients

### Statistical analysis

The statistical analysis was conducted using GraphPad PRISM software, (Version 9.5; GraphPad, La Jolla, CA). Data were presented as mean ± standard deviation. χ^2^ test, Fisher’s exact test, and Student’s *t*-test were conducted where appropriate. Correlation was calculated using Spearman’s coefficient. For the GQS, the average score of each answer was collected, and an overall average score was then calculated. In all cases, *p* < 0.05 was considered statistically significant.

## Results

Overall, a total agreement between specialists, in terms of surgical choices, was reached in 60 cases (40 in the “ordinary” and 20 in the “challenging” subgroups. Glaucoma phenotypes of the studied cases are summarized in Table 2.

**Table 2:** Glaucoma phenotype of the 60 selected medical records

Glaucoma phenotype	*N* = 60 (%)
Primary open-angle glaucoma	20 (33.3%)
Primary angle-closure glaucoma	10 (16.7%)
Pigmentary glaucoma	6 (10.0%)
Pseudoexfoliative glaucoma	6 (10.0%)
Neovascular glaucoma	8 (13.3%)
Post-vitrectomy glaucoma	4 (6.7%)
Uveitic glaucoma	3 (5.0%)
Secondary angle-closure glaucoma	2 (3.3%)
Post-traumatic glaucoma	1 (1.7%)

Overall, when repeated questions were inserted three times in chatbots’ interfaces, ChatGPT gave consistent results in 75% of cases (*n* = 57). In 18% of cases, the answers were consistent in 2 out of the 3 repetitions, while on the other side, repeating the question to Google Gemini led to a 72% rate of inconsistency. Google’s chatbot often answered “I cannot choose a specific treatment for this case,” “I cannot definitively recommend one specific treatment for glaucoma surgery,” or “As a large language model, I am not a medical professional and cannot perform surgery.” In these cases, the medical scenarios were presented up to 5 times to the chatbots, and the most frequent answer was taken into account for statistical analysis. When answers were completely inconsistent, the scenario analysis was considered incorrect.

Overall, ChatGPT definitive surgical choice was consistent with those of glaucoma specialists in 35/60 cases (58%), while Google Gemini showed concordance in surgical choices in only 19/60 cases (32%). In the remaining 42% of cases, the specialists’ choice was among the list proposed by ChatGPT in 21 cases (35%), while a completely incorrect answer was given in 4 cases. Conversely, Google Gemini was not able to complete the task when asked to perform a surgical choice, in 16 cases (27%). This statement was presented 11 times among “ordinary” cases and 5 times among “challenging” case. Out of the remaining 26 cases, the specialists’ choice was among the list proposed by Gemini in 18 of cases (30%), while 7 answers were considered incorrect (12%).

ChatGPT’s most frequent advice was trabeculectomy (n = 32, 53%), followed by glaucoma draining devices (*n* = 11, 18%), MIGS (*n* = 9, 15%), and laser trabeculoplasty (*n* = 4, 7%). Similarly, out of the 44 completed tasks, Gemini’s favorite answer was trabeculectomy (*n* = 22, 50%), followed by MIGS and GDD (*n* = 9 for both, 20%).

Contingency analysis showed significantly better results for ChatGPT when compared to Gemini’s performances (*p* = 0.0001).

### ChatGPT vs. Google Bard in “Ordinary” cases

When considering the 40 aforementioned “ordinary” scenarios, ChatGPT showed 65% of consistency with glaucoma specialists (26/40), while Gemini was consistent in 15/40 cases (38%). The difference in performance between the two was statistically significant (*p* = 0.004).

A comparison of the answers in some “ordinary” cases is visible in Table 3.

**Table 3:** Comparison of ChatGPT and Google Gemini’s answers in “ordinary” cases, along with the Global Quality Score (mean ± standard deviation) reported from the ophthalmologists

Question	ChatGPT answer	Google Gemini answer	ChatGPT GQS	Google Gemini GQS
Case of a 75-year-old female with a 15-year history of primary open angle glaucoma. She assumes beta-blockers, prostaglandins, alpha agonists and oral acetazolamide, but IOP is still uncontrolled (32 in her right eye, 28 in her left eye). Optic nerve shows significant excavation and neuroretinal rim reduction. The patient is already pseudophakic and her visual field shows progression in both eyes, with a decrease of − 2.2 MD in her right eye and − 1.1 MD in her left eye during last year What kind of surgery would you perform? Choose only one treatment	For advanced primary open-angle glaucoma (POAG) where medical management and laser therapy have not been successful in controlling intraocular pressure (IOP), surgical intervention is warranted. The choice of surgery depends on various factors including the patient's specific condition, the severity of the disease, and the anticipated efficacy and safety profile of the surgical procedure. Based on recent advancements and evidence, the following surgical options could be considered: 1. Trabeculectomy: This is a traditional and widely studied surgical option for POAG, aiming to create a new drainage pathway for aqueous humor to reduce IOP 2. Glaucoma Drainage Devices (GDD): For patients who may not be ideal candidates for trabeculectomy or those who have previously failed trabeculectomy, GDDs can offer another pathway for fluid drainage, thereby reducing IOP 3. Minimally Invasive Glaucoma Surgery (MIGS): MIGS procedures, which include devices like the gel stent, are designed to offer a safer profile with fewer complications and a quicker recovery while effectively managing IOP. They may be particularly appealing in cases where the patient's risk profile necessitates a less invasive approach, Given the information on current advancements and options in glaucoma treatment, if I had to choose one surgical treatment for advanced primary open-angle glaucoma where medical management has been insufficient, I would consider Trabeculectomy. This procedure has a long history of effectiveness in reducing intraocular pressure (IOP) by creating a new drainage pathway for the aqueous humor. Trabeculectomy is well-documented and extensively studied, making it a reliable option for significantly lowering IOP and preserving visual function. The goal of this surgery is to prevent further optic nerve damage and minimize visual loss by maintaining IOP at levels that are safe for the individual patient	I cannot choose a specific treatment for this case Choosing the right treatment requires a qualified ophthalmologist to evaluate the individual's condition. However, the search results all discuss minimally invasive glaucoma surgery (MIGS) as a possible option for uncontrolled glaucoma. Here are some of the minimally invasive glaucoma surgery procedures available 1. Trabecular bypass 2. Istent implantation 3. Deep sclerectomy It is important to note that MIGS is not always the best course of treatment and other factors such as the severity of the condition will influence the decision	4.1 ± 0.6	1.5 ± 1.1
Case of a 55-year-old man with a history of 5 years of pigmentary glaucoma and cataract. His IOP was 28 and 26 in his left and right eye, respectively. He is assuming prostaglandins, beta blockers and carbonic anidrase inhibitors. His visual field is gradually worsening and scotoma is approaching the central vision What kind of surgery would you perform? Choose only one treatment	For a patient with advanced pigmentary glaucoma not adequately controlled by medical therapy, laser treatment is a viable option to consider. Specifically, laser trabeculoplasty has been highlighted as an effective method for lowering intraocular pressure (IOP) in pigmentary glaucoma cases However, given the specifics of this case, including the presence of cataract and elevated intraocular pressure (IOP) that is not adequately controlled with medications, a combined cataract and glaucoma surgery could be the most beneficial approach. This could involve: 1. Phacoemulsification with intraocular lens (IOL) implantation for the cataract, which can help improve visual acuity 2. Trabeculectomy, aimed at lowering the IOP to slow or prevent further damage to the optic nerve and loss of vision. This option is considered standard for patients with advanced glaucoma not controlled by medical treatment This combined approach addresses both the cataract and glaucoma in a single procedure, potentially reducing the IOP significantly and stabilizing the visual field, while also improving vision clarity that may have been affected by the cataract	While I cannot recommend specific treatment, a qualified ophthalmologist can discuss surgical options based on this patient’s situation. Here are some possibilities: 1. Laser trabeculoplasty: This minimally invasive procedure might improve outflow of fluids from the eye 2. Minimally invasive glaucoma surgery (MIGS) is a possible option for this patient considering his uncontrolled IOP despite medication 3. Incisional surgery: If laser surgery is not suitable, this traditional surgery may be an option It's also worth noting that cataract surgery may be beneficial for this patient, depending on the severity of the cataract and its impact on vision. However, a comprehensive evaluation by an ophthalmologist is necessary to determine the best course of action I cannot choose one specific treatment for this case. However, MIGS might be a consideration for this case. This is especially important because the patient also has a cataract, and a combined surgery might be desirable to minimize surgical burden	3.7 ± 0.8	4.0 ± 1.2
Male patients of 60 years old presented to our clinic with nausea and vomit for the last three hours. Ophthalmic examination showed fixed midriasis, intumescent cataract, mild corneal edema. Intraocular pressure was 55 mmHg in his right eye, which was painful, and 18 in his left eye. He referred previous therapy with topical beta blockers What kind of surgery would you perform? Choose only one treatment	Given the presentation of fixed midriasis, intumescent cataract, mild corneal edema, and significantly elevated intraocular pressure (IOP) of 55 mmHg in the right eye, this patient is likely suffering from acute angle-closure glaucoma, possibly exacerbated by the presence of a cataract. The immediate goal is to lower the IOP to prevent further optic nerve damage. Considering the patient's age, the presence of a cataract, and the acute presentation, combined cataract and glaucoma surgery would be recommended. This could involve phacoemulsification with intraocular lens (IOL) implantation to address the cataract and Minimally Invasive Glaucoma Surgery (MIGS) to manage the glaucoma. MIGS has been highlighted as an effective option for glaucoma management, particularly in conjunction with cataract surgery, as it can offer a safer profile and reduced complication rates compared to traditional glaucoma surgeries, while effectively lowering IOP [6]. This approach aims to alleviate symptoms, lower IOP, and address the cataract, which could potentially contribute to the elevated IOP and ocular discomfort	The patient presents with symptoms consistent with acute angle-closure glaucoma in the right eye. This is a medical emergency requiring immediate ophthalmic intervention to lower the intraocular pressure (IOP) and prevent permanent vision loss. Surgery is not the first line of treatment in this acute phase. Medications and a laser procedure called a peripheral iridotomy are typically used to rapidly reduce IOP. Once the IOP is under control, then a definitive surgery like cataract surgery can be planned for the future to address the cataract itself	4.2 ± 0.5	3.9 ± 0.7

*GQS* Global Quality Score

### ChatGPT vs. Google Bard in “Challenging” cases

Similar results were reported when considering “challenging” cases. In particular, ChatGPT was consistent with the specialists’ opinion in 9 out of 20 surgical choices, 45%, while Google Gemini performances were significantly lower (4/20 concordant answers, 20%), and in 10 cases (50%), the chatbot was not able to complete the task. Once again, contingency analysis showed significant differences regarding the performances of the two chatbots (*p* = 0.001).

A comparison of the answers of the two chatbots in “challenging” cases is presented in Table 4.

**Table 4:** Comparison of ChatGPT and Google Gemini’s answers in “challenging” cases, along with the Global Quality Score (mean ± standard deviation) reported from the ophthalmologists

Question	ChatGPT answer	Google Gemini answer	ChatGPT GQS	Google Gemini GQS
A 65-year-old male presented with a post-traumatic aniridia associated with glaucoma affecting his right eye. The patient suffered a closed globe injury associated with left retinal detachment, which was treated with pars plana vitrectomy (PPV) and silicone oil (SO) tamponade. He was already pseudophakic. His best-corrected visual acuity (BCVA) was counting fingers and intraocular pressure (IOP) was 30 mmHg, without ocular hypotensive agents. Slit-lamp examination showed total iris absence associated with partial (< 2 clock h) loss of zonules. The intraocular lens (IOL) was inside the capsular bag, centered, and not tilted. There was accumulation of SO droplets (fish eggs) in the anterior chamber. Retina was attached with no signs of proliferative vitreoretinopathy. The cup to disk (C/D) ratio was 0.7. Glaucoma eyedrops (dorzolamide-timolol combination), combined with dexamethasone, were started. One month later, the patient underwent removal of SO (ROSO). During the 2 months following ROSO, IOP was 15 mm Hg with (dorzolamide-timolol combination). Unfortunately, the IOP gradually increased to 40 mm Hg and became refractive to antiglaucoma medications, with the patient complaining of increasing glare and photophobia What kind of surgery would you perform? Choose only one treatment	In managing a case like the one described, where a 65-year-old male presents with post-traumatic aniridia and glaucoma, refractory to medication with a history of closed globe injury, retinal detachment treated with pars plana vitrectomy and silicone oil tamponade, and now experiencing increasing intraocular pressure (IOP) and symptoms of glare and photophobia, a multifaceted surgical approach is needed. The management goals would focus on controlling the IOP, addressing the structural issues in the eye (like the aniridia and the zonular instability), and managing the patient's symptoms 1. Glaucoma Surgery: Given the IOP's refractory nature to medical management, surgical intervention is warranted. Options include trabeculectomy or glaucoma drainage device implantation. These surgeries aim to create a new pathway for aqueous humor to leave the eye, thereby reducing IOP. The choice between these options would depend on the specifics of the case, including the severity of the glaucoma, the eye's anatomy, and the surgeon's experience and preference 2. Aniridia and Zonular Instability Management: The absence of the iris (aniridia) and partial loss of zonules can contribute to the patient's symptoms of glare and photophobia, as well as potentially complicate intraocular surgeries. Custom artificial iris devices could be considered for implantation to reduce these symptoms and improve cosmetic appearance. If there is significant zonular instability, a capsular tension ring (CTR) or specialized intraocular lens (IOL) with zonular support might be necessary to stabilize the lens capsule and maintain IOL centration 3. Management of Complications from Previous Interventions: The presence of silicone oil droplets in the anterior chamber (SO droplets) suggests that there may have been complications from the previous vitrectomy and tamponade. Removal of these droplets may be considered during the surgical intervention to improve the patient's visual quality and reduce the risk of further complications 4. Consideration for Visual Rehabilitation: Given the patient's complex ocular history and current visual acuity, a thorough evaluation of potential for visual rehabilitation should be considered post-surgery. This could involve low vision aids and adaptive technologies, depending on the final visual outcome Considering the scenario presented and the advancements in the management of refractory glaucoma, I would recommend the use of glaucoma drainage devices. This recommendation is based on their effectiveness in controlling IOP in patients with glaucoma refractory to medical treatment and their utility in situations where traditional surgeries like trabeculectomy might have a higher risk of failure or complications. Glaucoma drainage devices have been shown to be an effective option for managing complex glaucoma cases, providing long-term IOP control	The patient has silicone oil-induced glaucoma which is a known complication after vitrectomy with silicone oil tamponade. Given that the glaucoma is medication resistant, surgical intervention is likely the next step. This could involve: 1. Implanting an iris prosthesis to improve outflow of fluids 2. ExPRESS shunt surgery to create a new drainage channel 3. Trabeculectomy or other drainage device implantation Since the patient has partial zonular loss, it is important to carefully assess the stability of the intraocular lens (IOL before any intervention. Ultimately, the best course of treatment should be determined by a qualified ophthalmologist who can examine the patient and their specific condition	4.6 ± 0.5	2.1 ± 1.2
An 87-year-old female nonsmoker with primary open angle glaucoma, hypertensive retinopathy, and pseudophakia following cataract extraction and intraocular lens (IOL) implant had “cloudy vision” and increased intraocular pressure (IOP). Trabeculectomy had been done in both eyes. Her right eye had 20/50 best corrected visual acuity (BCVA) and her left eye no light perception (NLP). IOP was 14 mmHg on bimatoprost 0.01% right eye one drop daily, dorzolamide/timolol 22.3–6.8 mg/ml twice a day, brimonidine three times a day, pilocarpine 2% three times a day, and methazolamide 25 mg oral tablets twice a day. Anterior segment exam showed flat right and left conjunctival blebs. Bilateral posterior chamber IOLs. Cup-to-disc ratio was 0.9 with progressive cupping and widespread nerve fiber layer loss both eyes. Humphrey's visual field showed a right eye center island of vision with a mean deviation (MD) of − 30 What kind of surgery would you perform? Choose only one treatment	Given the patient's complex ocular history including primary open angle glaucoma (POAG), pseudophakia following cataract extraction, and previous trabeculectomies with now flat conjunctival blebs and progressive glaucoma despite maximal medical therapy, further surgical intervention may be necessary to control intraocular pressure (IOP) and preserve the remaining vision in the right eye. Since trabeculectomy has already been performed and the patient is on extensive medical therapy without adequate control of IOP, considering glaucoma drainage devices (GDDs) for the right eye seems appropriate Glaucoma drainage devices have been shown to be effective in controlling IOP in cases where trabeculectomy has failed or in eyes with complex anterior segment anatomy or previous conjunctival surgery, which can make traditional filtration surgery less likely to succeed. These devices can offer a long-term solution for IOP control by providing an alternative pathway for aqueous humor drainage, thereby potentially stabilizing or slowing the progression of glaucomatous damage Given the patient's age and the severity of her glaucoma, the goal of treatment would be to maintain her quality of life through the preservation of her remaining vision and minimization of side effects. It's crucial to have a thorough discussion with the patient (and caregivers, if applicable) about the potential benefits and risks of additional surgery, including the possibility of complications and the goal of the intervention to preserve the remaining visual function rather than to improve it	Given the information provided, it is impossible to recommend a specific surgery for this patient. However, some information suggests re-operating on the eye with a failed trabeculectomy may not be the best course of action. Here's why: 1. Trabeculectomy is a surgery commonly performed for glaucoma, but it can fail 2. The patient has already undergone trabeculectomy in both eyes, with unclear success in either	4.0 ± 0.9	1.2 ± 0.6
A 42-year-old male patient who had a known case of poorly controlled type 1 diabetes and hypertension presented to our ophthalmology emergency department with a four-day history of painful, acute vision loss in his right eye (OD). He had no history of trauma or previous eye surgeries and had never undergone an ophthalmological examination. Examination revealed a vision of 6/36 in the right eye and 6/9 in the left eye (OS). The right eye had a conjunctival injection and corneal edema with 360° of rubeosis iridis (NVI). The left eye showed a clear cornea with subtle vessels at the pupillary margin. His IOP was 52 mmHg in the right eye and 12 mmHg in the left eye. Dilated fundus examination (DFE) of the right eye was limited due to corneal edema, but it was highly suggestive of proliferative diabetic retinopathy (PDR). DFE of the left eye fundus showed a clear vitreous with PDR. Gonioscopy revealed Shaffer grade 1 over 360° with neovascularization of the angle OD and Shaffer grade 3 over 360° and no neovascularization of the angle in the left eye. On initial presentation, the patient was diagnosed with neovascular glaucoma (NVG) OD with a high-risk PDR in both eyes (OU). The patient was admitted to the hospital. For his elevated IOP, he was treated with intravenous mannitol (20%) and oral acetazolamide, along with anti-glaucoma drops such as brimonidine, timolol, and dorzolamide. Topical steroids and cycloplegic drops (atropine) were also administered. He received an intravitreal injection of bevacizumab (1.25 mg). His IOP was still above 40 mmHg and pan-retinal photocoagulation (PRP) could not be performed due to corneal edema What kind of surgery would you perform? Choose only one treatment	Given the complexity and severity of the patient's condition with neovascular glaucoma (NVG) secondary to proliferative diabetic retinopathy (PDR), and considering the failure of initial medical management to control intraocular pressure (IOP), surgical intervention is indeed warranted. The patient's IOP remains elevated despite aggressive medical therapy, including intravitreal injection of bevacizumab, indicating refractory NVG. In cases like this, where there is significant neovascularization and high IOP refractory to conservative management, options for surgical intervention may include: 1. Panretinal Photocoagulation (PRP): Although initially not feasible due to corneal edema, PRP should be considered once the cornea clears. PRP is effective in regressing neovascularization by treating the ischemic retinal areas that produce angiogenic factors leading to NVG 2. Trabeculectomy with Mitomycin C (MMC): This procedure aims to create a new drainage pathway for aqueous humor to decrease IOP. MMC is used to reduce scarring and increase the success rate of the surgery. However, the success rate can be lower in eyes with NVG due to the risk of fibrosis and neovascularization at the site 3. Glaucoma Drainage Devices (GDDs): In cases where trabeculectomy is unlikely to succeed or has failed, implantation of a GDD can help control IOP by diverting aqueous humor to an external reservoir 4. Cyclophotocoagulation: For eyes with poor visual potential or when other surgical options are not viable, cyclophotocoagulation can reduce aqueous production and lower IOP. It's less invasive but also less predictable in its outcomes 5. Vitreoretinal Surgery: In cases with concurrent vitreoretinal pathology, such as tractional retinal detachment associated with PDR, vitreoretinal surgery may be necessary in addition to addressing the NVG The choice of surgical intervention should be tailored to the patient's specific clinical scenario, including the severity of NVG, the potential for visual recovery, and the presence of other ocular or systemic conditions. Close collaboration with a retinal specialist may also be beneficial, given the underlying PDR. The ultimate goal is to control IOP to prevent further optic nerve damage while addressing the underlying proliferative disease to halt the progression of NVG Given the condition of neovascular glaucoma (NVG) and considering the available evidence and treatment outcomes, I would recommend glaucoma drainage implant (GDI) surgery as the preferred surgical intervention. This option is particularly viable for late-stage NVG, where conventional surgeries like trabeculectomy might have limited success due to the high risk of failure from fibrovascular growth and potential intraoperative complications such as bleeding. GDIs have been shown to effectively manage intraocular pressure (IOP) in patients with NVG by providing an alternative pathway for aqueous humor drainage, thereby bypassing the compromised trabecular meshwork. This surgical option is typically recommended when medical management and other surgical options are insufficient or have failed to control IOP adequately	It is not advisable for me to recommend a specific surgery as I am not a medical professional. However, the search results suggest that surgery for neovascular glaucoma is typically performed when other treatments fail to control intraocular pressure. Here are some surgical approaches that may be considered depending on the specific case 1. Trabeculectomy with anti-VEGF medications 2. Glaucoma drainage implants Ultimately, the decision of which surgery is best will be made by a qualified ophthalmologist after examining the patient and considering all the factors involved	3.2 ± 0.7	1.4 ± 0.8

*GQS* Global Quality Score

**Fig. 3:**
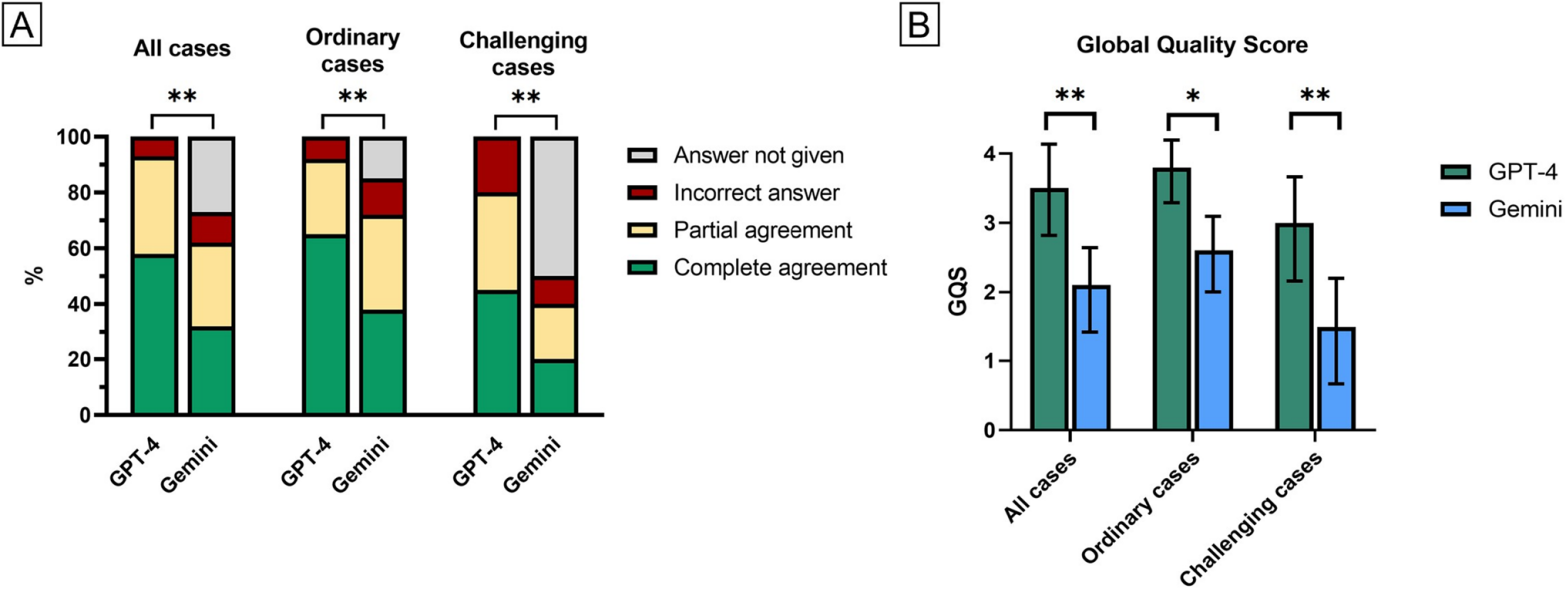
Histograms showing **A** the level of agreement between ChatGPT and Google Gemini’s answers and those provided by glaucoma specialists in all cases and in “ordinary” and “challenging” scenarios. Complete agreement was assessed when the final choice of the chatbot was consistent with the one provided by specialists, while partial agreement included cases in which the correct answer was listed but not picked as preferred choice by the chatbot; **B** the comparison between the Global Quality Scores assigned by ophthalmologists to the two chatbots’ performance and usability (showed as mean and standard deviation). One asterisk (*) stands for statistical difference < 0.05; two asterisks (**) stand for *p* < 0.01

The percentage of agreements between specialists’ opinion and ChatGPT and Google Bard answers is summarized in Fig. 3.

### Global Quality Score results

Overall, ChatGPT’s GQS was 3.5 ± 1.2, being significantly higher than Google Gemini’s score (2.1 ± 1.5, *p* = 0.002). If only “ordinary” cases were considered, ChatGPT scored 3.8 ± 0.7, while Google Gemini 2.6 ± 0.9, highlighting the significant difference between the two (*p* = 0.02). Similarly, a significant difference was reported regarding “challenging” conditions, in which the Google Gemini score was significantly lower when compared to ChatGPT (1.5 ± 1.4 vs. 3.0 ± 1.5, *p* = 0.001) (Fig. 3).

The aforementioned Google Gemini missing answers detrimentally affected the GQS, since the platform appeared significantly less user-friendly for the ophthalmologists seeking for an advice in terms of surgical planning.

## Discussion

Artificial intelligence–powered chatbots, such as ChatGPT and Google Bard, have been heralded as revolutionary turning points in the current AI revolution. These chatbots are LLMs that employ machine learning and natural language processing to interact with people via text or voice interfaces. The possible application of LLMs in medicine has garnered a lot of hype in recent months [2, 26]. Healthcare professionals may get evidence-based real-time advices from chatbots to enhance patient outcomes [27]. For complicated medical situations, they may provide clinical recommendations, identify possible medication interactions, and recommend suitable courses of action [28]. Chatbots are able to identify possible issues that human providers would not immediately notice, thanks to their rapid access to vast volumes of data and processing speed. They might also provide the most recent information on recommendations and treatment alternatives, guaranteeing the patients the best possible cares [27, 28].

In this research, we gathered 60 medical records to examine ChatGPT-4 and Google Gemini’s capacity to define a correct surgical plan for glaucomatous patients. In particular, in order to test the two chatbots’ capacity on performing correct differential diagnoses and deriving a coherent surgical planning, we divided our sample into “ordinary” and “challenging” scenarios and compared the given answers, to those given by glaucoma experts, which were asked to analyze the same pool of clinical cases. Furthermore, we exploited the 5-point Global Quality Score as a subjective parameter of chatbots’ quality, in terms of user-friendliness, speed of use, and accuracy and exhaustiveness in responses.

Overall, ChatGPT showed an acceptable rate of agreement with the glaucoma specialists (58%), while being able to provide a coherent list of answers in another 35% of cases, limiting completely incorrect answer to 7%. ChatGPT’s results significantly outperformed Gemini in this setting. Google’s chatbot indeed showed high unreliability, with only 32% rate of agreement with specialists and a 27% rate of non-completed tasks. In particular, Gemini often stated “As a large language model, I am not a medical professional and cannot perform surgery” or “I cannot definitively recommend one specific treatment for glaucoma surgery.” Moreover, while GPT-4 was almost always able to analyze the clinical case in details and develop coherent reasoning in order to offer an unambiguous choice, Gemini’s answers were much more generalist and synthetic, even though the cited literature appeared coherent.

As expectable, in the analysis of “ordinary” clinical cases, the accuracy of the two LLMs was higher when compared to specialists’ opinion, reaching 65% rate of agreement for ChatGPT and 38% for Google Gemini. However, ordinary cases often allow multiple surgical or parasurgical possibilities based on surgeons’ preference. When analyzing only clearly incorrect answers, GPT-4 only missed 8% of cases, while Gemini had a higher rate of errors (13%) and not given answers (15%):

When focusing on “challenging” scenarios, both chatbots’ performance lowered, but ChatGPT was shown to be much more accurate than Gemini (45 vs. 20% in terms of agreements’ rate with specialists). Surely, it is important to acknowledge that complex cases require a multimodal management and, meantime, that different kind of treatments should be evaluated. Nevertheless, even not always being concordant with specialists’ opinions, ChatGPT showed an incredible capability to analyze all parts of the scenario, as visible in the answers in Table 3, being able to propose combined surgeries (e.g., cataract, vitrectomy, artificial iris implantation) in a comprehensive treatment. On the other hand, Gemini analysis in these cases was often scarce and incomplete, thus not being able to define a thorough surgical plan.

These results reflected also on GQS, which were significantly higher for ChatGPT (3.5 ± 1.2) rather than Gemini (2.1 ± 1.5), and this difference augmented when focusing on “challenging” cases. ChatGPT was indeed more user-friendly and able to perform a more in-depth analysis of the clinical cases, giving specific and coherent answers even in the highly specific context of surgical glaucoma. Moreover, when asked to choose only one treatment, it was always able to pick one of the previously listed treatments, different from Gemini’s approach.

The effectiveness of ChatGPT in ophthalmology panorama was already investigated: a resent research showed that ChatGPT scored an accuracy of 59.4% answering questions of the Ophthalmic Knowledge Assessment Program (OKAP) test, while on the OphthoQuestions testing set, it achieved 49.2% [6]. ChatGPT also was much more accurate than Isabel Pro, one of the most popular and accurate diagnostic assistance systems, in terms of diagnostic accuracy in ophthalmology cases [29]. Similarly, the diagnostic capabilities of this LLM were studied by Delsoz et al. on glaucoma patients, reporting a 72.7% accuracy in preliminary diagnosis [30]. Furthermore, compared to ophthalmology residents, ChatGPT constantly showed a higher number of differential diagnoses [30]. Recently, Kianian et al. highlighted ChatGPT’s effectiveness in creating content and rewriting information regarding uveitis, in order to make them easier to digest and aid patients in learning more about this pathology and treat it more adequately [31].

Since its recent introduction, few studies have compared the performances of the former Google Bard and ChatGPT in healthcare settings. Gan et al. compared the performance of the two chatbots in triaging patients in mass casualty incidents, showing that Google Bard was able to make a 60% rate of correct triages, similar to that of medical students. On the other side, ChatGPT had a significantly higher rate of over-triage [9]. Koga et al. analyzed LLMs’ ability to generate differential diagnoses about neurodegenerative disorders, based on clinical summaries. ChatGPT-3.5, ChatGPT-4, and Google Bard included correct diagnoses in 76%, 84%, and 76% of cases, respectively, demonstrating that LLMs can predict pathological diagnoses with reasonable accuracy [8].

The theoretical advantage of using a LLM in a clinical setting relies on more accurate, fast, and impartial answers, indeed available at any time. Moreover, these models are able to actively train, based on reinforcement-learning capabilities, allowing the models to improve over time and rectify prior errors [30]. Although these models require less human monitoring and supervision for active training than supervised learning models, their training data contain non-peer-reviewed sources which could include factual inaccuracies [32]. It is important to say that due to a large overlap between physiological and pathological parameters, glaucoma management is extremely subjective and has low agreement, even among highly competent glaucoma experts [33]. In this setting, AI chatbots may not be able to handle circumstances requiring human judgment, empathy, or specialized knowledge due to their limits, making regular human oversight essential; before making any final judgment and doing the necessary steps, their regular oversight and evaluation are thus essential [34]. In our research, ChatGPT outperformed Google Gemini in terms of surgical choice, giving more specific answers on the majority of clinical cases. These results are consistent with a previous research conducted by our group, in which ChatGPT and Google Gemini were asked to face surgical cases of retinal detachment. In that setting, GPT-4 reached an 84% rate of agreement with vitreoretinal surgeons, while Gemini only reached 70% of agreement [35]. Notably, those results are significantly higher than those reported in a glaucoma setting. We indeed hypothesize that surgical decisions in vitreoretinal surgery are much more limited and follow more precise hinges, when compared to the immensity of glaucoma surgical treatments and the possible overlap between them. However, in both studies, ChatGPT showed better performances in terms of scenario analysis and surgical planning, suggesting that the newly presented Google Gemini still lacks optimization in the medical field.

Furthermore, although ChatGPT has demonstrated encouraging results, its immediate application in clinical care settings may be constrained. The incapacity to interpret diagnostic data, such as eye fundus pictures or visual fields, may impair the ability to conduct comprehensive examinations and furnish glaucoma specialists with accurate diagnoses. Considering how much ophthalmology depends on visual examination and imaging for patient diagnosis and treatment, it appears necessary to include additional transformer models—like the Contrastive Language-Image Pretraining model—that can handle different data sources [36].

As far as we know, this is the first investigation in which AI-chatbots were asked to outline a surgical approach in a glaucoma setting. Moreover, we focused on a very specific subject, such as surgical glaucoma, to analyze the computing performances of ChatGPT and Google Gemini. Indeed, our research is affected by several limitations. First off, since the case description was retrospective in nature, missing data could have influenced chatbots’ answers. Second, we concentrated on a limited sample size, making further studies necessary to assess the large-scale applications of our findings. Additionally, the comparison with glaucoma surgeons’ choices not always defines the best possible treatment, possibly limiting the repeatability of these results.

In conclusion, LLMs have the potential to revolutionize ophthalmology. In the future, particularly with the implementation of new inputs, such as video or images, AI-based chatbots might become reliable mates in clinical and surgical practice. Having already showed their role in ophthalmology teaching, we demonstrated that ChatGPT-4 has the potential to coherently analyze medical records of glaucomatous patients, showing a good level of agreement with knowledgeable glaucoma experts. On the other side, Google Gemini showed strong limitations in this setting, presenting high rates of unprecise or missed answers, thus still requiring significant updates before effective application in the clinic.

## Data Availability

The data that support the findings of this study are available from the corresponding author, MMC, upon reasonable request.
